# Patterns of diagnostic testing for oesophagogastric cancer-related symptoms in Australian primary care: a retrospective cohort study

**DOI:** 10.3399/BJGP.2024.0621

**Published:** 2025-02-18

**Authors:** Shaoke Lei, Brent Venning, Alison Pearce, Alex Lee, Jon Emery

**Affiliations:** 1 Department of General Practice and Primary Care, Melbourne Medical School, University of Melbourne, Melbourne, Australia; 2 Collaborative Centre for Genomic Cancer Medicine, University of Melbourne, Melbourne, Australia; 3 Daffodil Centre, University of Sydney, a joint venture with Cancer Council NSW, Sydney, Australia; 4 Sydney School of Public Health, University of Sydney, Sydney, Australia

**Keywords:** general practice, oesophageal neoplasms, stomach neoplasms, gastrointestinal diseases, diagnostic techniques and procedures, socioeconomic factors

## Abstract

**Background:**

Oesophagogastric (OG) cancer-associated symptoms are common in primary care, but most research has focused on patients with a confirmed OG cancer diagnosis, rather than those presenting with symptoms for the first time.

**Aim:**

To examine diagnostic testing patterns for upper gastrointestinal (GI) symptoms linked to OG cancer.

**Design and setting:**

A retrospective cohort study was undertaken, which used a linked primary care database. It included de-identified patients aged >55 years who presented with symptoms suggestive of OG cancer between 2008 and 2022.

**Method:**

The study analysed the proportion of patients who underwent pathology, imaging, referral, upper GI endoscopy, or a test of treatment. Differences across socioeconomic groups were also examined, along with the proportion of patients diagnosed with OG cancer.

**Results:**

The study cohort consisted of 44 402 patients, of whom 126 (0.3%) were diagnosed with OG cancer within 12 months of presentation. Reflux was the most common symptom (57%), followed by nausea (11%). Patients aged ≥75 years were less likely to be investigated or referred than those aged 56–64 years (odds ratio [OR] 0.59, 95% confidence interval [CI] = 0.56 to 0.62, *P*<0.001). Those from less disadvantaged areas were 1.4 times more likely to be investigated than people from the most disadvantaged areas (OR 1.44, 95% CI = 1.36 to 1.53, *P*<0.001). Patients on test-of-treatment medications were less likely to receive further investigation (OR 0.66, 95% CI = 0.63 to 0.69, *P*<0.001). Multiple symptoms and visits increased the likelihood of investigation (OR 2.77, 95% CI = 2.55 to 3.00, *P*<0.001).

**Conclusion:**

Significant variations in diagnostic testing could contribute to disparities in OG cancer outcomes.

## How this fits in

Oesophagogastric (OG) cancer-associated symptoms are common in primary care. Existing evidence has focused on patients with a diagnosis of OG cancer rather than those with symptoms presenting to general practice for the first time. Older patients (aged ≥75 years) and those from disadvantaged areas were less likely to be investigated while patients with multiple symptoms or visits were more likely to undergo pathology and/or imaging or specialist referral. Of 44 402 patients aged >55 years with upper gastrointestinal (GI) symptoms, 0.3% (*n* = 126) were diagnosed with OG cancer. A more systematic and equitable approach is required to investigate upper GI symptoms in primary care, to reduce variations in cancer outcomes.

## Introduction

Undifferentiated illness is a large part of a GP’s caseload, requiring careful judgement to balance necessary investigations for serious conditions, such as cancer, with avoiding over-investigation.^1^ This is particularly challenging with upper gastrointestinal (GI) symptoms, which are non-specific and linked to both benign conditions and cancers, such as oesophagogastric (OG) cancer.^2^ In Australia, GI issues account for nearly 10% of GP encounters,^3^ and while dysphagia is the strongest predictor of OG cancer, up to 50% of patients present with other symptoms such as pain, reflux, and weight loss.^4^

Despite the high prevalence of upper GI symptoms and the role of general practice in investigating OG cancers, limited Australian data exist on their management.^5^ One study found that for every 100 unresolved GI symptom encounters GPs requested pathology in 82% of cases, prescribed medication in 34%, made referrals in 16%, and ordered imaging in 22%.^6^ International research highlights the influence of different health systems on investigation methods, limiting cross-country comparisons.^7^

In Australia, concerns have been raised about upper GI endoscopy overuse, with rising rates despite stable cancer incidence.^8^ Upper GI endoscopy rates are higher in affluent areas, despite a higher prevalence of upper GI symptoms and OG cancer incidence in disadvantaged groups.^8^

Understanding diagnostic testing patterns for upper GI symptoms is crucial for identifying potential gaps in care, ensuring timely diagnosis, and optimising the use of healthcare resources. This study aimed to examine testing patterns for common upper GI symptoms associated with OG cancer using a linked Australian primary care dataset.

## Method

### Data sources

Data were obtained from the Victorian Comprehensive Cancer Centre Alliance Data Connect programme.^9^ This study drew on the Primary Care Audit, Teaching and Research Open Network (Patron) database, which is a general practice electronic health record database containing de-identified information from GP encounters, covering about 1.5 million patients from >130 Victorian general practices from 2008–2022.^10^ The Centre for Victorian Data Linkage (CVDL) carried out the data linkage using a system of linkage identifiers to combine personal records from Patron, the Victorian Admitted Episodes Dataset (VAED), and the Victorian Cancer Registry (VCR) data.^11^ VAED contains data on admitted patient episodes to public and private hospitals in Victoria from 2008–2022.^12^ VCR collected records for patients diagnosed with cancer in Victoria from 2008–2022.^13^

### Symptoms related to OG cancer

The cancer-related symptoms of interest were derived from the Australian government-endorsed Optimal Care Pathways and published risk assessment tools and include gastro-oesophageal reflux, anorexia, dysphagia, nausea, dyspepsia, abdominal bloating, epigastric pain, unexpected weight loss, and anaemia.^14,15^ Symptom information is contained within a ‘reason for encounter’ field in the patient’s electronic health record. The reason for encounter can be extracted from custom lists unique to each electronic medical record software or can be entered by the clinician using free text. There is no standardised format used across all primary care medical record systems. To account for the variability in symptom descriptions, additional synonyms were devised and incorporated to capture alternative expressions of the symptoms of interest. This approach increases the likelihood of identifying relevant symptoms and enhances the accuracy of symptom extraction and analysis. Supplementary Box S1 details the symptoms included for each symptom group, along with exclusion terms. The approach to review free-text entries is outlined in Supplementary Figure S1.

### Study cohort

**Figure 1.**
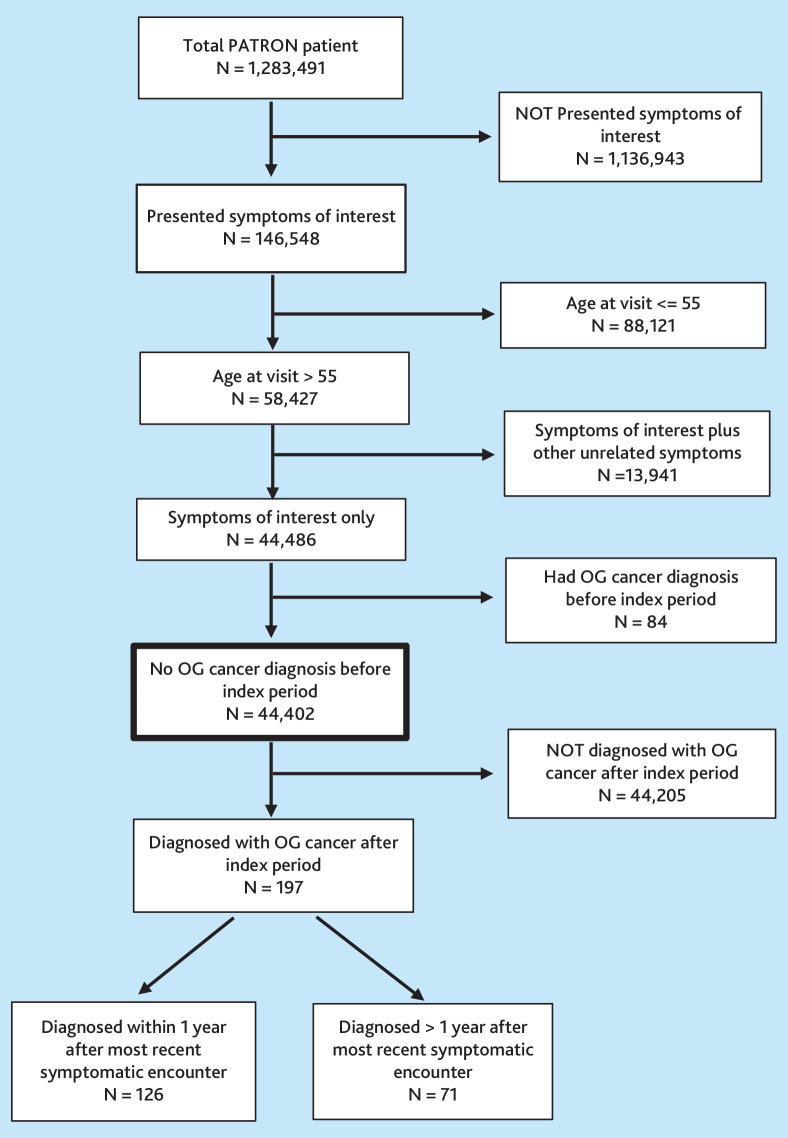
Flowchart outlining the steps involved in selecting the final patient cohort for the study. OG = oesophagogastric.

Figure 1 outlines the steps in selecting the study’s final patient cohort. To define the patient cohort with possible OG cancer symptoms, the Patron database was searched for relevant symptoms in patients aged >55 years. The most recent GP encounter was identified where any symptom of interest was present. Then the preceding 12 months were reviewed before this encounter to check for other relevant symptoms. The analysis was limited to symptoms within the 12 months before the most recent consultation to ensure temporal relevance between symptoms and subsequent OG cancer diagnosis. If multiple symptoms were present within the index period, it was assumed they were related to the same illness. If a patient had multiple symptoms, such as epigastric pain and nausea at the same encounter, they were recorded together as a combination and were not counted separately. Encounters containing multiple unrelated issues within the same encounter were excluded owing to difficulty determining which investigations corresponded to which issues.

A total of 58 427 patients aged >55 years were identified based on their presenting symptoms at the time of their visit. After excluding cases with unrelated multiple symptoms and patients previously diagnosed with OG cancer, 44 402 patients were included in the analysis.

Using VCR data, patients diagnosed with OG cancer were identified but any patients previously diagnosed before their most recent symptomatic presentation were excluded. Demographic data obtained from Patron at the time of symptomatic encounter included patient age, sex, remoteness area,^16^ Index of Relative Socioeconomic Disadvantage (IRSD) quintile,^17^ and smoking status.

### Investigations

A list of pertinent investigations was compiled to capture tests relevant to OG cancer and other potential differential diagnoses for the presenting symptoms. Tests typically requested at the sub-specialist level, such as oesophageal manometry or pH monitoring for upper GI symptoms, were omitted. Primary care investigations were limited to those conducted at the time of the symptom encounter of interest. Any investigations ordered before or after this encounter were excluded, as their relevance to the presenting complaint could not be determined. The investigations extracted from the primary care data are listed in Supplementary Box S2. Since referral information is recorded as free text in Patron, relevant referral letters were only captured from the same symptomatic encounter. It was determined whether patients underwent upper GI endoscopy at a Victoria hospital within 12 months after their general practice visit using the VAED dataset.^^12^^

### Medications used as a test of treatment

'Test of treatment' refers to assessing a patient’s response to a particular therapy to confirm or exclude diagnoses.^18^ Within Patron, prescriptions often include a patient’s regular medications, such as antihypertensives, for example, which may not be directly related to their primary complaint. This generates large amounts of data, which may not be pertinent to the research question. To manage this volume of data, a selection of relevant medications were extracted from the *Australian Medicines Handbook*, which may have been used as tests of treatment for the presenting upper GI symptoms (see Supplementary Box S3).

### Identifying patients with OG cancer

The final cohort of patients were linked to the VCR using *International Statistical Classification of Diseases, 10th Revision, Australian Modification* (ICD-10-AM) codes C15 and C16, converted from VCR’s ICD0 codes. Cancers diagnosed within 12 months from the last symptomatic presentation were included, and cancers by symptoms with patient number (*n*) <5 were excluded as per CVDL’s requirement.

### Statistical analysis

The frequency of symptom presentations in general practice were calculated, and sample characteristics were described for patients who had and had not undergone investigation. Categorical data were compared using a χ^2^ two-sample test. A multiple logistic regression model was employed to explore factors associated with diagnostic testing or referral, where variable selections were conducted using backward elimination with Akaike information criterion.

The proportion of patients diagnosed with OG cancer within 12 months of presentation was described by calculating the ratio of total cancer cases per symptom to the total number of patients presenting with that symptom and the ratio of total cancer cases to the entire cohort number.

The time to diagnosis was estimated using median and interquartile ranges for the time to diagnosis in days from the first symptomatic encounter.

All datasets underwent cleaning, management, and analysis using R (version 4.3.1), which were conducted securely on the online platform Victorian Data Access Linkage Trust.

## Results

### Patient characteristics

Reflux was the most frequent presenting symptom, representing 57% of total presentations, followed by nausea, which accounted for 11% of total presentations (see Supplementary Figure S2). Table 1 presents the demographic characteristics of patients stratified by whether diagnostic testing or referral was requested. Female patients outnumbered male patients, with no notable differences in testing proportions between sex. Younger patients (aged 56–64 and 65–74 years) were more likely to have had diagnostic testing requested. Patients in major cities were investigated more than patients in inner and outer regional areas but not remote areas. Patients in less disadvantaged areas were more likely to undergo diagnostic testing and/or referral than those in the most disadvantaged areas.

**Table 1.** Characteristic table comparing patients who were and were not investigated (including pathology, imaging, and upper gastrointestinal endoscopy) or referred

Factor	Investigation (testing or referral)				*P*-value
	Yes		No		
	*n*	%	*n*	%	
**Sex**					
Female	10 863	40	16 249	60	0.02
Male	6739	39	10 547	61	
**Age group, years**					
56–64	5973	44	7694	56	<0.001
65–74	5887	43	7841	57	
≥75	5742	34	11 265	66	
**Remoteness**					
Major cities of Australia	9460	41	13 530	59	<0.001
Inner regional Australia	6799	39	10 859	61	
Outer regional Australia	1295	36	2323	64	
Remote Australia	14	45	17	55	
**IRSD quintile**					
1 (most disadvantaged)	3891	37	6725	63	<0.001
2	3901	36	6852	64	
3	2909	39	4489	61	
4	3041	44	3890	56	
5 (least disadvantaged)	3812	44	4761	56	
**Medication for test of treatment**					
No	9667	46	11 461	54	<0.001
Yes	7935	34	15 339	66	
**Complexity**					
Single encounter and single symptom	12 349	35	22 698	65	<0.001
Multiple encounters and single symptom	3288	55	2726	45	
Multiple encounters and multiple symptoms	1745	61	1110	39	
Single encounter and multiple symptoms	220	45	266	55	
**Smoking status** ^a^					
Non-smoker	9231	41	13 175	59	0.41
Ever-smoker	7191	41	10 439	59	

^a^Ever-smoker includes both current and ex-smokers. IRSD = Index of Relative Socioeconomic Disadvantage.

### Variation of investigations and test-of-treatment strategies

**Figure 2.**
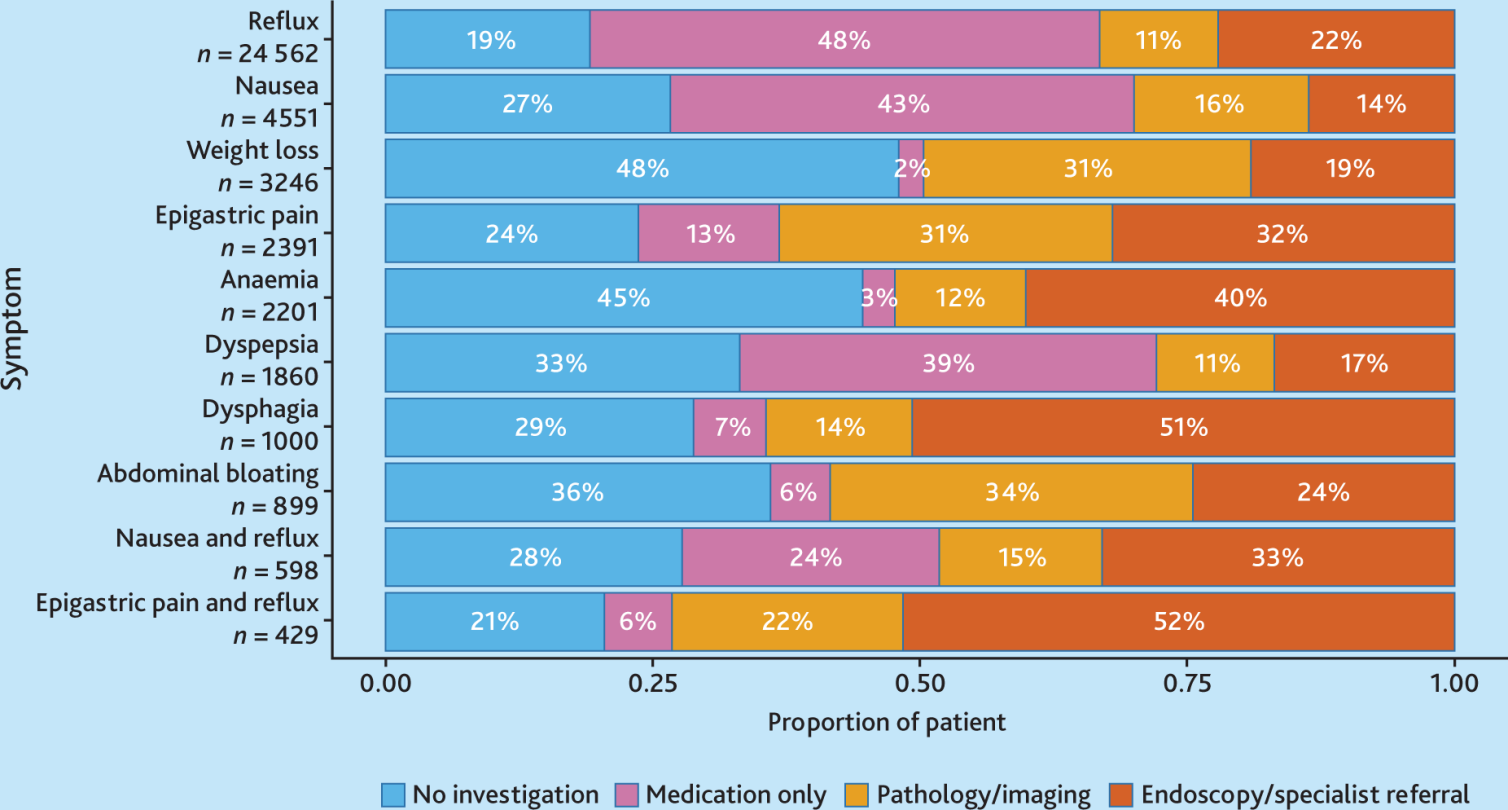
Proportion of patients with oesophagogastric (OG) cancer-related symptoms managed by either no investigation, medication only, pathology or imaging, or upper gastrointestinal endoscopy or specialist referral. Symptoms are listed in order of the 10 most common OG cancer symptoms across patients.

The investigation type across symptoms is described in Figure 2. More than half of patients presenting with dysphagia (51%) and epigastric pain plus reflux (52%) underwent either upper GI endoscopy or were referred for disease specialist assessment. Pathology and/or imaging tests were most frequently requested for patients with symptoms of abdominal bloating (34%), epigastric pain (31%), and weight loss (31%). Symptoms most managed with medication included reflux (48%), nausea (43%), and dyspepsia (39%). No investigation was performed for almost half of the patients presenting with weight loss (48%) and anaemia (45%).

**Figure 3.**
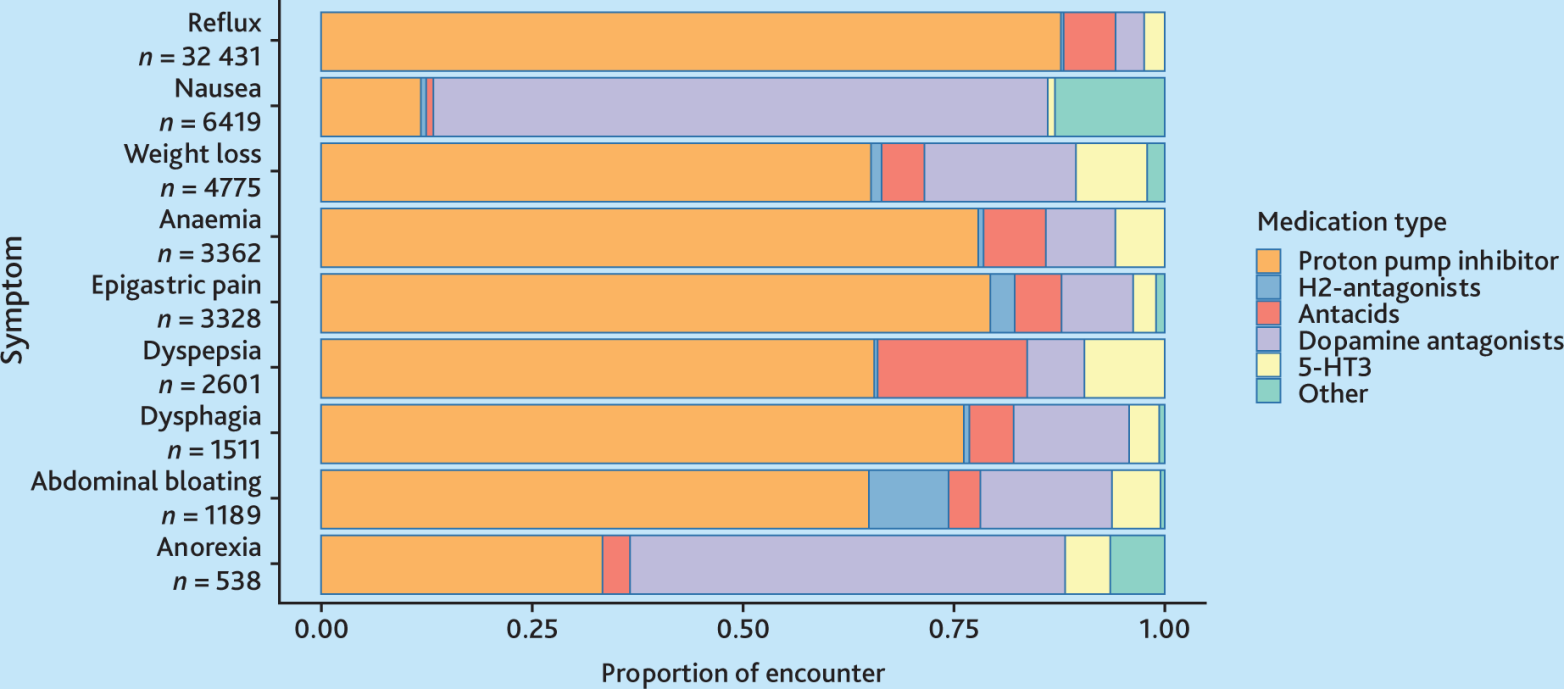
Variation of medication prescriptions for oesophagogastric (OG) cancer-related symptoms. Symptoms are listed in order of the nine most common OG cancer symptoms by reason for encounter.

**Figure 4.**
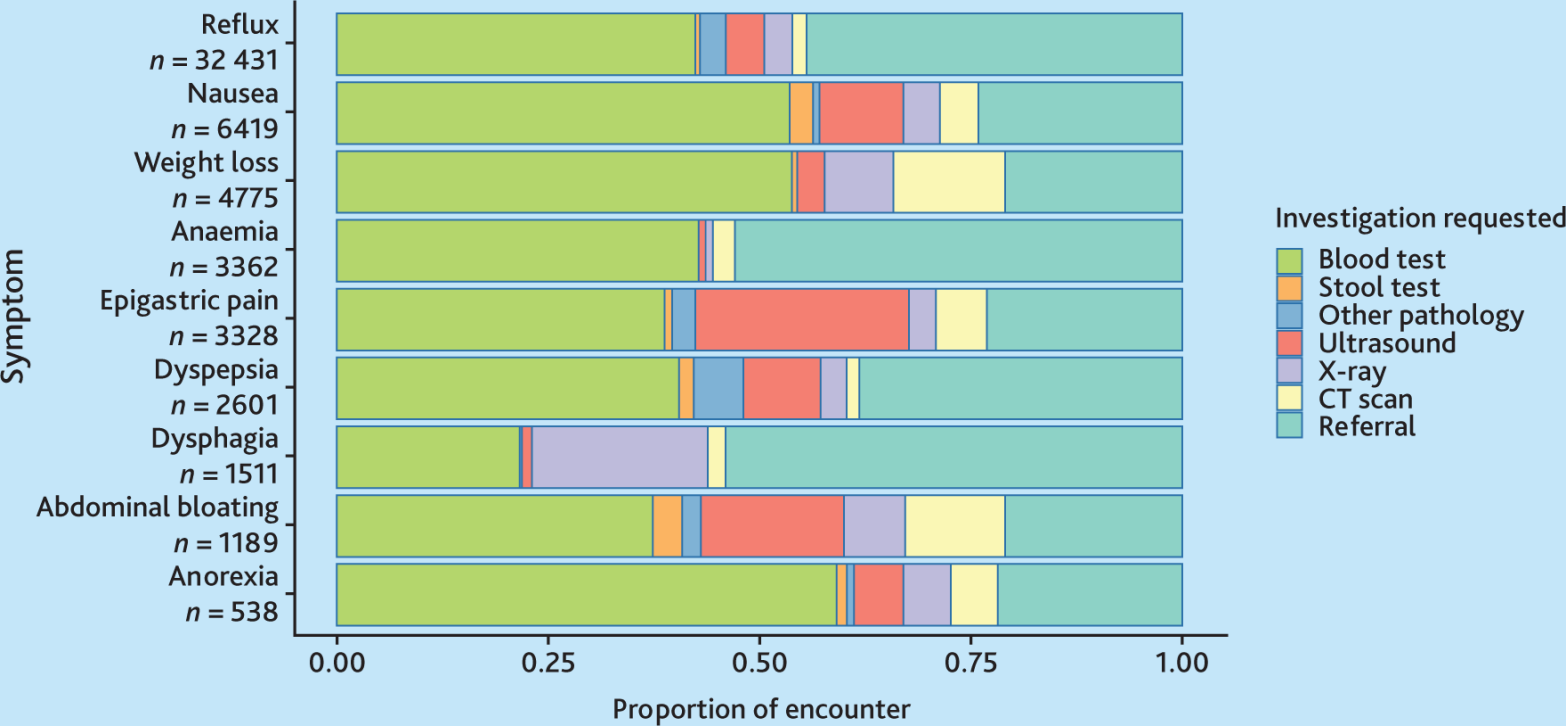
Variation of investigations ordered for oesophagogastric (OG) cancer-related symptoms across the 9 most common patient encounters. Symptoms are listed in order of the 10 most common OG cancer symptoms by reason for encounter. CT = computed tomography.

Proton pump inhibitors (PPIs) were the predominant medication prescribed for most symptoms, except for nausea and anorexia, where dopamine antagonists were preferred (Figure 3). Blood tests were the most frequently requested investigations by GPs (Figure 4). Ultrasound was the most common imaging modality. However, for red-flag symptoms, such as dysphagia, more than half of the presentations resulted in referrals (Figure 4).

### Factors associated with diagnostic testing

Table 2 presents the results of the regression model of the relationship between diagnostic testing or referral request and sociodemographic variables. Female patients were more likely to be tested than male patients (odds ratio [OR] 1.04, 95% confidence interval [CI] = 1.00 to 1.09, *P* = 0.043). Older patients, especially those aged ≥75 years (OR 0.59, 95% CI = 0.56 to 0.62, *P*<0.001), were less likely to undergo further testing or clinical referral than those aged 56–64 years. A significant association was observed between socioeconomic status (SES) and investigation, with patients from the least disadvantaged areas (IRSD 5) being approximately 1.4 times more likely to be investigated than those from the most disadvantaged areas (IRSD 1) (OR 1.44, 95% CI = 1.36 to 1.53, *P*<0.001). When broken down by investigation type, more disadvantaged individuals were more likely to undergo investigations in primary care. In contrast, advantaged patients were more likely to be referred to specialists and/or undergo upper GI endoscopy (see Supplementary Table S1). Patients prescribed test-of-treatment medications were less likely to undergo further investigations (OR 0.66, 95% CI = 0.63 to 0.69, *P*<0.001) (Table 2). Patients with multiple symptoms and/or multiple presentations were more likely to be investigated with pathology and/or imaging or specialist referral. For instance, the odds of investigation or referral for repeated presentations with >1 symptom were 2.77 times higher than for single presentations with only one symptom (OR 2.77, 95% CI = 2.55 to 3.00, *P*<0.001).

**Table 2.** Results of multiple logistic regression model showing the odds ratio of factors associated with diagnostic testing or referral

Variable	OR	95% CI	*P*-value
**Sex**			
Male	Reference		
Female	1.04	1.00 to 1.09	0.043
**Age, years**			
56–64	Reference		
65–74	0.95	0.90 to 1.00	0.04
≥75	0.59	0.56 to 0.62	<0.001
**IRSD quintile**			
1 (most disadvantaged)	Reference		
2	1.01	0.95 to 1.06	0.863
3	1.07	1.00 to 1.14	0.037
4	1.35	1.27 to 1.44	<0.001
5 (least disadvantage)	1.44	1.36 to 1.53	<0.001
**Medication for test of treatment**			
No	Reference		
Yes	0.66	0.63 to 0.69	<0.001
**Complexity**			
Single encounter and single symptom	Reference		
Multiple encounters and single symptom	2.16	2.04 to 2.29	<0.001
Multiple encounters and multiple symptoms	2.77	2.55 to 3.00	<0.001
Single encounter and multiple symptoms	1.34	1.12 to 1.61	0.002

IRSD = Index of Relative Socioeconomic Disadvantage. OR = odds ratio.

### OG cancer diagnosis

The details of patients diagnosed with OG cancer, overall and categorised by symptom, can be found in Supplementary Table S2. In summary, from a cohort of 44 402 patients, 126 patients were diagnosed with OG cancer within 12 months, representing 0.28% of the total cohort. Among those diagnosed with OG cancer, reflux was the commonest symptom, with 35 out of 24 562 patients (0.14%) diagnosed with OG cancer. Dysphagia was the most strongly predictive of OG cancer, with 27 out of 1000 patients (2.70%) diagnosed with cancer. Weight loss was observed in 3246 patients, with 11 (0.34%) diagnosed with OG cancer. Epigastric pain and anaemia were each present in slightly over 2200 patients, with nine cases of cancer diagnosed in each group (0.38% and 0.41%, respectively). The median time to diagnosis from first visit for all symptoms was 35 days (interquartile range [IQR] 15–84 days), ranging from 13 days (IQR 12–24 days) for epigastric pain, 17 days (IQR 9–34 days) for dysphagia, and 76 days (IQR 28–227 days) for anaemia.

## Discussion

### Summary

This study represents the largest examination to date, to the authors’ knowledge, of the investigative patterns for symptoms associated with OG cancer in primary care. Pathology and/or imaging tests were most frequently requested for approximately one-third of patients presenting with symptoms of abdominal bloating (34%), epigastric pain (31%), and weight loss (31%). Symptoms most commonly managed with medication included reflux (48%), nausea (43%), and dyspepsia (39%). PPIs were the predominant medication prescribed for most symptoms.

Approximately half of the patients presenting with dysphagia and epigastric pain accompanied by reflux underwent either upper GI endoscopy or were referred for specialist assessment. Just over half of the patients presenting with symptoms of dysphagia were referred to specialist care for further investigation. Notably, patients from more advantaged backgrounds were more likely to undergo investigation, despite the lower incidence of OG cancer in areas with higher SES.^19^

Less than 3% of patients presenting with dysphagia were diagnosed with OG cancer within 12 months of presenting symptoms, underscoring the weak predictive nature of even so-called red-flag symptoms in primary care.

### Strengths and limitations

This study has several strengths. An inclusive approach was adopted by examining testing patterns for all patients with OG cancer symptoms, not only those with confirmed diagnoses. Using a clinical dataset provides robust, real-world data from clinical practice. By leveraging a linked primary care dataset, tests were examined both within and outside general practice settings. With a large sample of 44 402 patients, the study has strong statistical power to identify associations between patient characteristics and diagnostic outcomes.

The study aimed to create a cohort of symptomatic patients with clear links between each symptom and its investigation. When multiple unrelated issues are listed for an encounter, it becomes difficult to determine which investigations correspond to which symptoms. For example, if an encounter includes 'nausea', 'diabetes', and 'low mood' with various investigations ordered, it is unclear whether these tests are for each specific clinical code. To avoid this confusion, such encounters were excluded, although this resulted in a more selective cohort and may have excluded some patients diagnosed with OG cancer and may have biased the cohort toward a ‘healthier’ profile.

Using the reason for encounter as a method to assess symptoms has limitations, as it relies on accurate recording and may not capture every issue discussed during the consultation. Including free-text reasons helps reduce bias. The data are limited to general practices in Victoria, excluding encounters from practices outside the dataset. While key demographic factors were adjusted for, other confounders, such as clinician experience, patient preferences, and comorbidities, were not considered. It was beyond the scope of this study to examine the influence of comorbidities, but it is acknowledged that this may affect the propensity to investigate. Lastly, the study does not assess patient outcomes or the effectiveness of diagnostic strategies in detecting OG cancer.

### Comparison with existing literature

Blood tests were the most frequently ordered investigations, followed by ultrasounds and computed tomography (CT) scans, highlighting the predominant use of non-invasive diagnostic methods in primary care. The presence of multiple symptoms significantly heightened the likelihood of undergoing investigation, suggesting clinician recognition of the increased risk associated with multiple symptom presentations.^15^

Clinical guidelines recommend urgent investigation of clinical features such as dysphagia or anaemia owing to association with serious conditions such as cancer and gastrointestinal bleeding.^14,20,21^ Only 51% of dysphagia patients were referred for further investigation in the present study. This might be explained by variations in clinical practice or in how dysphagia is recorded within medical records. Despite applying exclusion criteria to omit pre-existing or oropharyngeal dysphagia, electronic medical records often group various swallowing disorders under a single label. This may have led to the inclusion of cases of non-oesophageal dysphagia in the present study cohort.

For the majority of single symptoms, the proportion of patients diagnosed with OG cancer was slightly lower than previously identified positive predictive values,^15^ but remained <1%. The most notable difference was observed with dysphagia: 2.7% of patients with dysphagia were diagnosed with OG cancer compared with approximately 5% in a primary care, case-control study.^15^ The approach in the present study differed in that the present study examined a large, undifferentiated cohort with a much larger sample size.

In the present study cohort, a significant number of patients underwent upper GI endoscopy, although few were diagnosed with oesophageal or gastric cancer. Of 44 402 individuals, around 14% had an upper GI endoscopy, with 126 diagnosed with OG cancer. While much debate on overuse focuses on patients aged <55 years, the present findings show low detection rates even in older symptomatic patients.^8,22^ This low detection rate carries economic implications given the high cost of upper GI endoscopy. Improving patient selection through better risk stratification could help reduce unnecessary testing.

The study found patients aged ≥75 years were less likely to receive further testing or referral despite the increase in cancer incidence rates in this age group. A recent systematic review of 54 studies involving 230 729 participants found that increasing age is associated with prolonged diagnostic intervals or deferred decisions to investigate cancer symptoms.^23^ The presence of frailty, comorbidities, and cognitive impairment were the most important factors that resulted in uncertainty in decisions involving older adults.^23^

The present study findings also reveal a disparity in diagnostic approaches by SES. Disadvantaged patients were more likely to be tested in primary care, while advantaged patients were more often referred for specialist care and upper GI endoscopy. Although much research has focused on how SES affects cancer symptom appraisal and help-seeking, little has examined its impact on clinician testing behaviour.^24,25^ One study found no clear link between readiness to investigate and GP demographics or practice characteristics.^26^ Clinicians may tailor diagnostics based on patients' access barriers, or patients with higher SES may be more likely to advocate for referral. The greater accessibility of upper GI endoscopy for privately insured patients likely contributes to lower use in disadvantaged areas.

### Implications for research and practice

Future research should clarify the factors behind disparities in testing and referrals, such as physician decision making, patient health literacy, healthcare access, and broader healthcare policies. It is recommended that safety netting, particularly for patients undergoing watchful waiting or trials of treatment, be implemented to help mitigate risk.^27^ Safety-netting advice should include a clear timeframe for reassessment, documentation of this timeframe, specific symptoms to monitor, and written guidance to support communication.^27^ In the Australian context, strategies such as reducing co-payments, ensuring full funding for radiology services, and decreasing waiting times for publicly funded upper GI endoscopy should be prioritised to address socioeconomic disparities.^28–32^ These measures can help improve diagnostic access for disadvantaged populations and support earlier detection of OG cancer.

## Data

The de-identified data analysed are not publicly available, and data used for this study can be shared subject to appropriate ethical approvals. All authors had full access to all of the data (including statistical reports and tables) related to the study.
